# A Fair Contribution Measurement Method for Federated Learning

**DOI:** 10.3390/s24154967

**Published:** 2024-07-31

**Authors:** Peng Guo, Yanqing Yang, Wei Guo, Yanping Shen

**Affiliations:** 1 School of Computer Science and Technology (School of Cyberspace Security), Xinjiang University, Urumqi 830046, China; guopeng@stu.xju.edu.cn; 2 Key Laboratory of Application Innovation in Emergency Command Communication Technology Ministry of Emergency Management, Ministry of Emergency Management Big Data Center, Beijing 100013, China; 3 School of Information Engineering, Institute of Disaster Prevention, Beijing 101601, China

**Keywords:** federated learning, Shapley value, contribution measurement, Non-IID

## Abstract

Federated learning is an effective approach for preserving data privacy and security, enabling machine learning to occur in a distributed environment and promoting its development. However, an urgent problem that needs to be addressed is how to encourage active client participation in federated learning. The Shapley value, a classical concept in cooperative game theory, has been utilized for data valuation in machine learning services. Nevertheless, existing numerical evaluation schemes based on the Shapley value are impractical, as they necessitate additional model training, leading to increased communication overhead. Moreover, participants’ data may exhibit Non-IID characteristics, posing a significant challenge to evaluating participant contributions. Non-IID data have greatly affected the accuracy of the global model, weakened the marginal effect of the participants, and led to the underestimated contribution measurement results of the participants. Current work often overlooks the impact of heterogeneity on model aggregation. This paper presents a fair federated learning contribution measurement scheme that addresses the need for additional model computations. By introducing a novel aggregation weight, it enhances the accuracy of the contribution measurement. Experiments on the MNIST and Fashion MNIST dataset show that the proposed method can accurately compute the contributions of participants. Compared to existing baseline algorithms, the model accuracy is significantly improved, with a similar time cost.

## 1. Introduction

In recent years, with the rapid development of machine learning, there have been great changes in various fields. ML, which is one of the most important areas of artificial intelligence, makes it possible to learn a model or a pattern of behavior for a given machine to perform tasks. ML algorithms allow us to process input data using appropriate patterns to generate output data. That is, we can identify and extract patterns from large amounts of data to build learning models [1], serving for predictive modeling and decision-making. In a distributed architecture, it is possible to use consensus mechanisms to manage data consistency [2]. Traditional machine learning methods usually require centralized collection and storage of large-scale data, and then training on a central server or in the cloud. As the public pays increasing attention to data privacy protection, the problem of data silos has become more severe, making the deployment of collective intelligence technology more challenging. Federated learning (FL), as a new training paradigm for artificial intelligence models dealing with data silos, has gained widespread attention in the past few years [3]. FL extends the training process of machine learning from a single device to multiple devices or compute nodes, enabling parallel processing and model synchronization through the distributed nature of data and computation. This shift can improve training efficiency, scalability and robustness, and provide better solutions in terms of privacy protection.

The performance of the federated learning (FL) system is heavily reliant on sustained participation from local users, high-quality data inputs, and authentic local training. User recruitment is a key aspect of federated learning, which includes attracting appropriate participants to join the FL system and effectively managing their participation behavior and contributions [4]. Naturally, not everyone is eager to share their data truthfully without any incentive. Hence, for a viable alliance, all participants should contribute high-quality data and be periodically rewarded accordingly [5]. However, a critical challenge lies in determining a fair compensation mechanism for these local data providers. Effective incentive mechanisms are invaluable in crowdsensing to stimulate the enthusiasm of strategic users [6]. Fairness means rewarding those who provide high-quality data and actively engage in federated learning [7] while punishing participants for bad behavior [8]. A fair return mechanism encourages participants to contribute high-quality data and perform local training in the long run, whereas an unfair allocation scheme may lead to participant misbehavior or permanent withdrawal. Existing federated learning (FL) platforms, such as the Federated AI Technology Enabler (FATE) [9], assume the system already has a stable participant group and does not need to attract more data providers. However, this precondition may not be satisfied in practice, especially when the participants are business organizations. Furthermore, since servers typically have limited knowledge of local users, the global model can be easily poisoned if the server blindly integrates all local models without proper evaluation. Therefore, it is necessary to design a fair contribution measurement method, which also serves as the customer evaluation plan, to quantify the contribution of participants, achieve fair distribution more effectively, and stimulate participants’ enthusiasm.

With the high development of information technology in the era of big data, all kinds of devices generate data, and the generation of data increases exponentially. Mobile devices are the main source of data generation, some of which have spatial characteristics, such as regional mobility, that have a high degree of randomness [10]. However, effective implementation of federated learning often depends on the availability of broad and diverse datasets that have traditionally been considered independent and equally distributed (IID) [11]. Assuming that the data have the same probability distribution and are independently distributed (IID) is a classic setup for machine learning. However, this is unrealistic: the composition and properties of things and our daily lives are heterogeneous, non-independent, and equally distributed [12]. The problem may be even more pronounced in federated learning. In federated learning, the challenge of heterogeneity often needs to be addressed, which refers to the Non-IID (non-independent and identically distributed) challenge caused by the imbalanced data distribution related to data labels and data quantities [13]. Existing literature suggests that compared to IID data, the Non-IID data may lead to significantly lower accuracy in federated learning [14].

Non-IID problems exist significantly in federated learning. This issue is due to biased labeling preferences at multiple clients and is a typical setting of data heterogeneity [15] and can be called category distribution heterogeneity. Intra-client category distribution heterogeneity (class imbalance) refers to the distribution of the amount of data between classes (i.e., class distribution) in the client being different from the uniform distribution. The greater the allocation gap, the more severe the imbalance [16]. For example, some clients may have a large number of samples belonging to class A, but a small number of samples belonging to class B, while other clients may have the opposite. This kind of dataset heterogeneity is common in federated learning, because different clients usually collect data locally, and these data may have label preferences or specific data collection methods. However, the existing contribution measurement methods of federated learning often ignore the impact of class distribution differences on participant contributions, resulting in inaccurate contribution results.

This paper proposes a more accurate method to measure the contribution of participants in federated learning that alleviates the influence of data distribution heterogeneity on contribution measurement, thereby improving the accuracy of contribution measurement and encouraging more participants to join federated learning.

The main contributions of this paper are as follows:

1. In the process of contribution measurement using Shapley, gradient multiplexing is performed: we use gradient approximation to reconstruct the model, which saves a lot of time. The reconfigurable model can be used to evaluate its performance more easily.
2. Considering the heterogeneity of data distribution, a new aggregate weight is used to mitigate the impact of data heterogeneity on contribution measurement and improve the accuracy of contribution measurement.
3. We propose a novel metric for measuring participant contributions in federated learning. By utilizing new aggregation weights, this method effectively mitigates the issue of data heterogeneity and enables a fairer assessment of participants’ contribution levels in the federated learning process.

This work is organized as follows: Section 2 describes the relevant background; Section 3 introduces the relevant work; Section 4 describes the formula definition and experimental method in detail; Section 5 describes the main experiments and results. Finally, in Section 6, the conclusions and future work are summarized.

## 2. Background

This section provides three main backgrounds: non-IID data, Shapley value, and federated learning. The federated learning approach uses an ML model shared by collaborative learning while protecting user privacy. Non-IID data refers to heterogeneous data with random characteristics. Heterogeneity of category distribution, one of the typical categories in non-IID, is a common problem in federated learning, which is often seen in the inconsistent label distribution among participants. The Shapley Value calculates the contribution of each participant by considering all possible combinations of parties where the contribution of a party is determined by the expected marginal gain in the value of the data when that party joins the federation [17]. The Shapley value scheme is intuitive, easy to understand, and ensures a fair assessment of each participant’s individual contribution, and it is widely used in current federated contribution assessment.

### 2.1. IID and Non-IID DATA

By statistical definition, IID means that the random variable data have the same probability distribution and are independent of each other. That is, the data are more homogeneous [18]. In other words, when the samples in the dataset are independent and drawn from the same distribution, we refer to it as IID data. In this case, each sample is sampled independently from the same data distribution. For example, if we have a dataset containing pictures of cats and dogs, and the labels for each sample are evenly distributed (i.e., the same proportion of cats and dogs), then the dataset is IID. In machine learning, it is commonly assumed that the data are independent and identically distributed.

When the samples in the dataset do not meet the conditions of independence and the same distribution, we call it non-IID data. As the training data on each client are collected based on the local environment and usage patterns, there can be significant variations in the size and distribution of the local datasets among different clients [19]. Consequently, the samples in the dataset may exhibit varying distributions or correlations. For instance, let us consider a dataset used for handwritten digit recognition, where the distribution of samples contributed by different clients may vary.

In federated learning, the IID or non-IID characteristics of data have a significant impact on the performance of model aggregation and global models. IID data assume that all clients have the same data distribution, which simplifies and enhances the reliability of model aggregation. Non-IID data, on the other hand, pose significant challenges, as the data distribution may vary among different clients. When the private dataset between clients is not independent and identically distributed (non-IID), the local training objective is inconsistent with the global training objective, which possibly causes the convergence speed of FL to slow down, or even not converge [20].

### 2.2. Shapley Value

The Shapley value (SV), named after Lloyd Shapley, is a well-established concept in cooperative game theory that aims to distribute the total profits generated by coalitions of players [21]. The Shapley value calculates the contribution of each participant to the utility of all possible coalitions they can be a part of and assigns a unique value to each participant [22]. It distributes the benefits among participants by considering all possible ways of cooperative arrangements, ensuring that each partner receives a fair reward based on their individual contributions. In a cooperative game, players can cooperate to generate a payoff. These participants may be individuals, organizations, or any other entity working together. The Shapley value is designed to address the challenge of fairly distributing rewards in cooperative games.

In cooperative games, multiple participants collaborate to achieve a common goal or generate value. The fundamental concept of the Shapley value is to quantify the contribution of each participant to the overall outcome, taking into account the order in which participants join the cooperative process. More specifically, the Shapley value represents the average marginal contribution of each participant towards the final outcome.

Consider a cooperative game involving n players. For a given player i, we consider all possible coalitions that player i can form with other players. The Shapley value is a method to distribute the payout among the players (the features) fairly. It evaluates all possible combinations of features, determining each feature’s contribution to the difference in the model’s prediction when included against when excluded [23]. The Shapley value is computed by aggregating the contributions of player i across all possible coalitions and taking a weighted average.

### 2.3. Federated Learning

The concept of federated learning was first proposed by a research team at Google in 2016 as a distributed machine learning paradigm to protect privacy [24]. In their research, the team introduced a distributed machine learning approach that enables model training using data scattered across different participating devices while preserving data privacy. The study aimed to address the privacy and security concerns associated with centralized storage of large-scale datasets in traditional centralized machine learning methods. In federated learning, each participant holds their own local data and conducts model training on their respective devices. They then transmit only the updated model parameters to a central server for aggregation. This approach mitigates the challenges associated with centralized storage and transmission of datasets, thereby reducing privacy risks and data transfer costs. Federated learning is extensively employed in various scenarios, including mobile devices, IoT devices, edge servers, etc., to accomplish tasks such as personalized recommendations, speech recognition, image classification, and more. Federated learning, as a novel distributed collaborative learning paradigm, has been widely applied in many scenarios and has successfully trained better models by sharing the private data of multiple clients without leaking privacy [25]. Due to these advantages, federated learning has greatly facilitated data collaboration and sharing among participants, addressing people’s concerns over data privacy.

**Figure 1:**
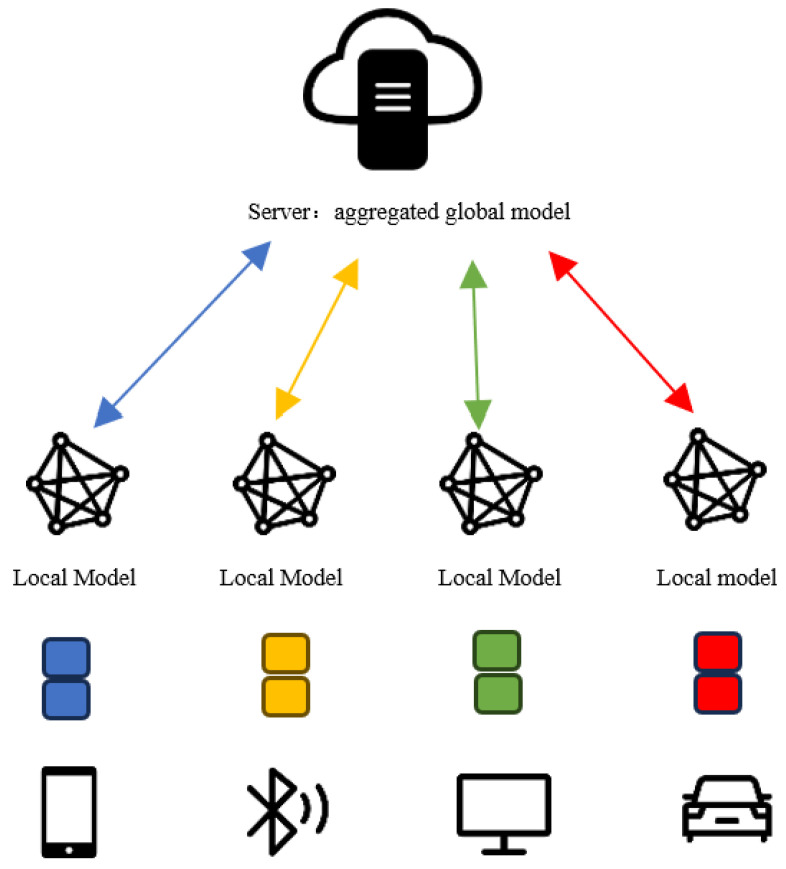
General federated learning structure diagram.

The FL system enables multiple parties to collaborate on learning tasks while keeping their data local to ensure security. In scenarios where strong privacy is essential, parties may need to be isolated from one another to prevent communication [26]. Federated learning is not merely a component of machine learning but rather a distributed machine learning framework that focuses on a data management process for sharing data among multiple clients while preserving privacy. Figure 1 llustrates the working principle of generic horizontal federated learning.

An illustrative example of federated learning is the word prediction task in the input method of smart devices. While performing this task, it is essential to protect users’ privacy and minimize communication congestion. Rather than transmitting private user data to a central server, training the predictor in a distributed manner is more reasonable. During this task, the smart device engages in periodic communication with the central server to acquire the global model. In each communication epoch, the selected smart device employs its local data for training and transmits the local model to the server. Following model aggregation, the server disseminates the updated global model to other device subsets. The process of continuous iterative training takes place between the server and the participating devices in federated learning until the model converges [27].

Participants establish communication with a trusted central server to exchange relevant information, including models, gradients, and more. Nonetheless, participants acquire this information based on their local data, which may exhibit non-IID (non-independent and identically distributed) characteristics. Moreover, due to simultaneous communication from multiple parties, there is a potential risk of communication congestion.

## 3. Related Work

Federated learning is a distributed framework that enables multiple clients to collaborate using their local data to train a shared model while maintaining data privacy and preventing data leakage [28]. In Ref. [29], the authors present the theoretical concepts of federated learning and apply them to develop a machine learning model using a vehicle’s basic safety information (BSM) dataset. The model is created through the on-board vanet network over the Internet of Vehicles (IoV) to ensure privacy during misconduct detection.

In current deep learning paradigms, local training or the Standalone framework tends to result in over-fitting and thus poor generalizability. This problem can be addressed by distributed or federated learning (FL) that leverages a parameter server to aggregate model updates from individual participants. However, most existing distributed or FL frameworks have overlooked an important aspect of participation: collaborative fairness [30]. This situation is inherently unfair. In practice, the contribution levels of participants vary for various reasons, such as the quality and quantity of data held by different participants. Federated learning is a collaborative machine learning framework involving multiple participants who maintain their training datasets locally. Evaluating the data contribution of each participant is one of the crucial challenges in federated learning [17]. Implementing a fair measurement method for participant contributions in federated learning ensures fairness among participants and rational resource allocation, establishes a basis for model selection, and encourages active participation in the federated learning process. This helps build a healthy, sustainable federal learning ecosystem. The work by Wang et al. [17] provides a detailed description of the challenges encountered in federated learning and presents common methods for measuring participant contributions, including leave-one-out and Shapley value.

Leave-one-out: The leave-one-out method (LOO) is widely used in machine learning tasks for cross-validation [31]. It is an intuitive data valuation method that measures a data point’s contribution by how much a model’s accuracy will lose after removing it [32]. Based on the idea of LOO, we can use the marginal loss in the value of the participant combination after excluding a certain participant as the contribution of that participant to the federation [33]. Unlike the individual method, the LOO method fully follows the participant combination data value measurement paradigm; that is, the contribution assessment and data value measurement problems are orthogonal. However, the LOO method only considers the marginal benefit brought to the federation by a certain participant when all other participants are fully retained, and this way of specifying the participant to join the federation last to evaluate the contribution also has fairness issues. For example, when there are multiple participants holding the same but highly valuable data for the federation, removing any one of the participants holding those data will not have a significant impact on the federation’s test accuracy, and these participants will be assessed as low-value, but at the same time, removing these participants will greatly reduce the performance of the federation. By eliminating the need for model retraining, this approach improves computation efficiency. Nevertheless, empirical experiments have demonstrated that LOO fails to capture the relative utility between any two samples [34], leading to undervaluation of individual contributions.

Shapley value: intuitive, easy to understand, to ensure fairness in assessing the individual contributions of each participant, the Shapley value (SV) is widely employed in current federal contribution assessment. Ghorbani et al. [32] introduce Data Shapley, which applies the concept of Shapley value (SV) to the data valuation problem. The SV of a data sample represents the average of its marginal contributions to the model considering all possible joining orders of the samples. Under this approach [32], the authors proposed a truncated Monte Carlo Shapley algorithm, which was implemented through random sampling arrangement and truncation of unnecessary sub-model training and evaluation, thereby reducing unnecessary model evaluations and significantly saving operational resources. Song et al. [7] argue that the existing contribution measurement scheme based on the Shapley value is not suitable for federated learning due to the additional model training involved, which incurs high costs. In order to solve this problem, the intermediate results of the federated learning training process are used to approximate the reconstruction of the model and reduce the additional model training process. When calculating the Shapley value, the challenge of $O(2^{n})$ permutations is unavoidable. If the number of participants increases significantly, implying a large value of n, it becomes impractical to enumerate all $2^{n}$ data combinations and compute all marginal contributions precisely. In the literature [34], the idea of the K-subset is adopted to address this problem. A stratified sampling method is used to randomly select each participant subset of possible size, and the occurrence time of size K is strictly recorded. The participant’s Shapley value is reconstructed to his expected contribution to a K-sized subset with random cardinality. Wei et al. [35] introduced a truncated multi-round (TMR) method in their paper, which is an improvement over the MR algorithm. It considers the accuracy of each round and assigns higher weights to training rounds with higher accuracy when performing the weighted averaging. It uses a decay factor to ignore the weights of the round-level $\phi^{\left(t\right)}$ in the last few rounds and only constructs and evaluates the models that have an effective influence on the final result.

Although federated learning (FL) offers an appealing framework for addressing the “data silos” challenge and decomposing a machine learning task into a collaborative effort, it faces several practical obstacles. On one hand, existing studies make the optimistic assumption that all mobile devices unconditionally contribute their resources [36]. However, this assumption is unrealistic and unfair to the parties involved. On the other hand, concerning training effectiveness, data distributed among different local participants are often non-independent and non-identically distributed (non-IID) [37]. Zhu et al. [38] believe that the presence of non-IID data inevitably results in a decline in FL accuracy, primarily due to the bias introduced by non-IID data into local model weights. The presence of heterogeneous data affects the convergence speed and learning ability of the model. Lyu et al. [39] introduced the federal average (FedAvg) model, a practical approach for joint learning of deep networks based on iterative averaging. FedAvg is frequently employed as an aggregation model, known for its robustness to non-IID data. Wang et al. [40] proposed a FedNova framework that reweights the target system’s heterogeneity to mitigate the non-IID problem. Hsu et al. [41] introduced the FedAvgM model, which stabilizes FL training using momentum on the server side to suppress oscillations through the continuous accumulation of gradient history. FedFTG [42] (Zhang et al.) fine-tunes the global model using hard samples to correct model drift after aggregation. FedGen [43] (Zhu et al.) addresses the issue of knowledge distillation requiring a proxy dataset by learning a feature generator from the local label distributions uploaded by clients to assist local training. This method allows the knowledge obtained from knowledge distillation to constrain local updates, enhancing client performance on non-IID data. However, these methods demand significant computational power from the server, resulting in much higher computational costs. In our proposed Shapley value-based contribution measurement method, the target data heterogeneity is reweighted based on local differences, alleviating the non-IID problem by adjusting the aggregation weights of the participants. Our method is computationally simple and performed locally, with participants not uploading any private information. Therefore, our approach does not impose additional computational burdens on the server, making the computational cost negligible.

## 4. Materials and Methods

Federated learning, as a distributed learning approach, can alleviate network congestion, prevent privacy leaks, and reduce the consumption of computational and communication resources. However, in federated learning, the size of the model parameters updated by training on local devices (which can reach billions) can reach tens of megabytes. Therefore, there may be a bottleneck when aggregating the model parameters in the parameter server [44]. In the current federated learning contribution evaluation schemes, evaluating individual utility through changes in model outputs often requires retraining multiple ML models, which poses a significant challenge to the aggregation server. The server must allocate additional computational resources for model retraining, which in turn affects the convergence speed of the models. Currently, the widely adopted algorithm for global model updates on the server is the FedAvg algorithm [45]. FedAvg, which performs weighted averaging based on the data volume from participants, is a common and effective method in federated learning. However, according to the Pareto principle, especially in cases of data heterogeneity, using only the one-dimensional data volume to obtain the aggregated global model is often insufficient [46]. The heterogeneity in data distribution not only affects the model’s accuracy but also impacts the evaluation of participants’ contributions. To solve these problems, we propose a fair contribution measurement scheme based on the Shapley value. Our proposed scheme not only avoids the need for additional sub-model retraining, greatly saving computational resources, but also mitigates the adverse effects of data distribution heterogeneity.

### 4.1. Contribution Measurement Method Based on the Shapley Value

As an example of horizontal federated learning, which we show in Figure 1, suppose *N* = {1,2,⋯,n} clients participating in federated learning have local private datasets $D_{i}$, *i*∈{1,2,⋯,n}. Suppose that federated learning requires *T* iterations to achieve a convergent model. In each epoch t ∈{1,2,⋯,T}, participant *i* downloads the global model $M^{\left(t\right)}$ from the server. It utilizes its local data for model training, resulting in a local model $M_{i}^{\left(t+1\right)}$. Subsequently, participant *i* sends the updated sub-model to the server, which calculates the corresponding gradient $\Delta_{i}^{\left(t+1\right)}$ based on the uploaded sub-model:

(1) $$\Delta_{i}^{\left(t+1\right)}=M_{i}^{\left(t+1\right)}-M^{\left(t\right)}$$

On the server side, it will collect each sub-model, calculate the update gradient of the corresponding participant, store it, and then perform an aggregation operation, which is the FedAvg algorithm in study [36] to update the global model. Its specific operations are as follows:

(2) $$M^{\left(t+1\right)}=M^{\left(t\right)}+\sum_{i}\frac{|D_{i}|}{|D_{N}|}\Delta_{i}^{\left(t+1\right)}$$

where $|D_{i}|$ is the size of training data $D_{i}$ of participant *i*, and $|D_{N}|$ is the sum of data owned by all participants.

Data Shapley value [32], ϕ (*N*,*U*), can be used to evaluate the contribution by each participant. It is defined as:

(3) $$\phi_{i}\left(N,U\right)=\sum_{S\subseteq N\backslash \{i\}}\frac{U\left(M_{S\cup \{i\}}\right)-U\left(M_{S}\right)}{\left(\begin{array}{c} n-1\\ \left|S\right| \end{array}\right)}$$

Here, *U* represents a utility evaluation function that measures the predictive performance of the learned model on an independent test set. In this paper, model accuracy is employed as the evaluation metric. The function is executed on the server, leveraging its higher computing power to efficiently obtain results without impacting the model’s runtime. The Shapley value is attractive for contribution evaluation problems because it satisfies some desirable axiomatic properties. We summarize the following common axioms [47]:

- Group Rationality: The value of the entire dataset is completely distributed among all users, i.e., $\phi \left(D\right)=\sum_{i\in D}\phi (i)$.
- Fairness: (1) Two users who are identical with respect to what they contribute to a dataset’s utility should have the same value. That is, if user *i* and *j* are equivalent in the sense that: i.e., ∀S⊆N\{i,j}, if U(S∪{i})=U(S∪{j}), then i=j; (2) Users with zero marginal contributions to all subsets of the dataset receive zero payoff: i.e., if U(S∪{i})=U(S), then i=0 for all ∀S⊆N\{i}.
- Additivity: The values under multiple utilities sum up to the value under a utility that is the sum of all these utilities: i.e.,∀i∈N, $\phi \left(U_{1}+U_{2},i\right)=\phi \left(U_{1},i\right)+\phi \left(U_{2},i\right)$, where $U_{1}$$U_{2}$ are two utility tests.

### 4.2. Gradient-Based Model Reconstruction

When evaluating contributions using Formula (3), additional sub-model reconstruction is often involved when evaluating $U(M_{s})$. To address this issue, we employ a gradient-based approach to reconstruct the sub-model in federated learning (FL): Suppose that in epoch *t*, we need to reconstruct the sub-model $M_{s}^{t}$ for performance evaluation, which can be defined as follows:

(4) $$U\left(M_{s}^{t}\right)=U\left(M^{t-1}+\sum_{i\in s}\frac{|D_{i}|}{|D_{s}|}\Delta_{i}^{\left(t\right)}\right)$$

where $M^{t-1}$ is the global model from the previous round, and $\Delta_{i}^{\left(t\right)}$ is the gradient information collected by the server in the current round. To evaluate the performance of the sub-model $M_{S}$, we only need to use the global model from the previous round and the gradient information from the subset of participants *S* in the current round to reconstruct the sub-model $M_{S}$ by Formula (4).

There is a clear difference between our approach and the current one: when calculating the Shapley value and needing to evaluate model $M_{s}$, the common approach is to retrain the model. The server would require all participants, except participant *i*, to perform local training again, and then aggregate the relevant information to reconstitute an $M_{s}$ for evaluation. This process consumes a significant amount of computational resources on the server. In our method, this step is replaced by reconstructing the sub-model using the previous round’s FL model and the current gradient information. Therefore, this method addresses the issue of needing extensive sub-model retraining to calculate SV in federated learning, greatly saving computational resources and accelerating the efficiency of SV computation.

### 4.3. Build a New Aggregate Weight

Currently, the commonly used algorithm for updating the global model on the server is the FedAvg algorithm [36]. However, this algorithm may have certain limitations. During the aggregate weight calculation process, the algorithm assigns weights solely based on the dataset sizes, neglecting the impact of data distribution heterogeneity. The presence of heterogeneous data weakens the contribution level of participants to the global model, which makes the results of contribution measurement inaccurate. To alleviate this issue, previous studies have mainly focused on regularization techniques for local models or fine-tuning the global model, overlooking the adjustment of aggregation weights, often assigning weights solely based on dataset sizes [15]. Considering the impact of data distribution heterogeneity on the model, it may be more reasonable to incorporate it into the calculation of aggregate weights in order to mitigate its effects.

Hence, our approach involves quantifying the issue of data distribution heterogeneity and incorporating it into the computation of updated aggregate weights. First, we assume a global category distribution $F_{G}$ on the server: A global category distribution is defined as one where each data category is evenly distributed, and the number of categories is consistent. This assumption promotes fairness among categories and enhances the generalization ability of the global model. Based on this assumption, we can calculate the local category distribution $F_{i}$ for each participant. During the global model aggregation phase, participants only upload their respective local models. For security reasons, local class distribution information is not uploaded. Subsequently, we can readily calculate the discrepancy between each participant’s distribution and the global distribution on the server side, denoted as $F_{k}$. In this study, we employ the Kullback–Leibler (KL) divergence to quantify the difference between these distributions:

(5) $$F_{\left(k=i\right)}=D_{\mathrm{KL}}\left(F_{i}∥F_{G}\right)=\sum_{i}P\left(F_{i}\right)\log\frac{P(F_{i})}{P(F_{G})}$$

**Figure 2:**
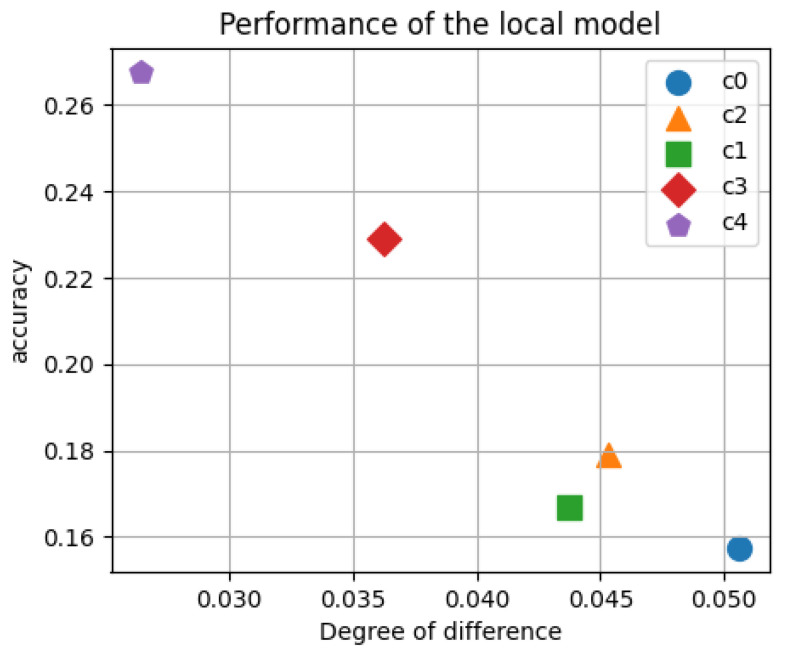
Impact of category distribution differences.

We studied the performance of local models on the test set for participants with different local category distribution differences, as shown in Figure 2. From the figure, it can be observed that participants with larger differences have poor performance in their local models. This poorly performing model will greatly affect the global model.

According to Formula (2), the weight of the size of the dataset in the formula is defined as $N_{k}$. That is to say: $N_{k}=\frac{|D_{i}|}{|D_{N}|}$. Using the previously calculated data distribution difference value $F_{k}$ for each participant, we can incorporate it into Formula (2) to obtain a new aggregate weight, denoted as $D_{k}$:

(6) $$D_{K}\propto N_{K}-a*F_{k}+b$$

Here, k=i, and *a* and *b* represent two constants. By utilizing Formula (6), we establish a relationship between the aggregate weights and the dataset size $N_{k}$ as well as the distribution of participant categories $F_{k}$. By formulating the aggregate weights, we can assign smaller weights to participants with higher difference levels, thereby mitigating the impact of heterogeneous data on the model. We design the aggregation weights of the participants reasonably to obtain an optimal weight, which can positively impact the experiment. The aggregation weights are closely related to the hyper-parameters *a* and *b*. By adjusting the values of *a* and *b*, we can assign an optimal aggregation weight to each participant to achieve the best performance. We will discuss the values of *a* and *b* in detail in the next section to obtain an optimal aggregation weight.

### 4.4. New Aggregate Function

Based on the preceding research, we can redefine Formula (2) by incorporating Formula (6) with the new aggregate weights, thus determining our final aggregate function.

(7) $$M^{t+1}=M^{\left(t\right)}+\sum_{i}D_{k=i}\Delta_{i}^{\left(t+1\right)}$$

For participants with large differences in local category distribution, the performance of the local model trained with local data is poor compared to participants with smaller differences in local category distribution. If the server directly uses this poor gradient information to aggregate the global model, it will inevitably affect the performance of the global model, leading to inaccurate SV measurement results. Our method calculates the local category distribution difference $F_{k}$ for each participant in the early stages of federated learning. In the federated aggregation stage, the server no longer uses only the proportion of the participants’ data as the aggregation weight, but also considers incorporating $F_{k}$ into the aggregation weight to mitigate the impact of the differences in the category distribution of the participants. Specifically, in the aggregation function of Formula (7), the aggregation weight for each participant can be dynamically adjusted: we can assign larger aggregation weights to participants with more data and smaller local category distribution differences, as the gradient information they upload is beneficial to the global model being trained; for participants with large local category distribution differences and small data volumes, we assign a smaller aggregation weight to reduce their impact on the global model, thereby improving the performance of the global model. Through Formula (3), we can also find that, with other metrics unchanged, the performance of the global model is significantly improved due to the greatly reduced impact of heterogeneous participants on the global model, which further increases the SV of the participant.

**Figure 3:**
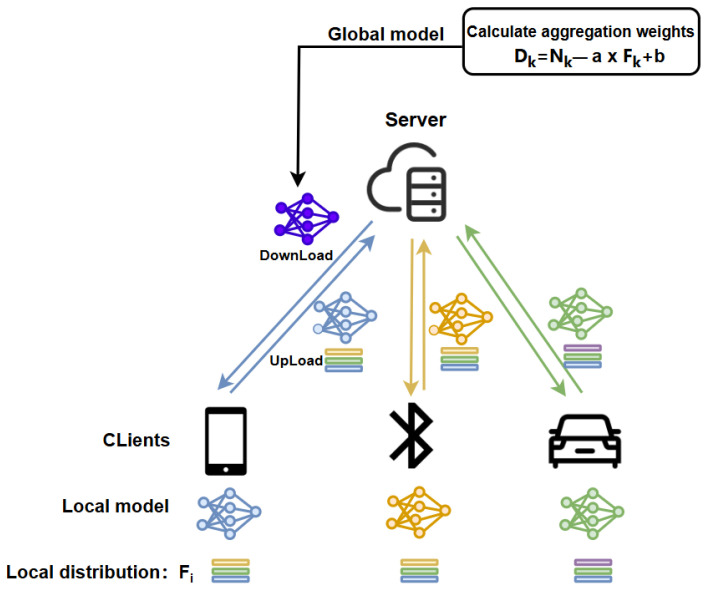
Modified federated learning structure diagram.

Based on the aforementioned modifications, we have established the model structure of this paper, which is illustrated in Figure 3.

During the model aggregation process, participants only upload their local models and their respective class distribution differences $F_{k}$. To protect privacy, the local category distribution $F_{i}$ is still saved locally and is not uploaded. Participants can easily calculate the difference between the local distribution and the global distribution based on $F_{G}$. The trusted server can then compute new aggregation weights $D_{k}$ based on $F_{k}$ and use them to update the global model, which is subsequently distributed to all participants.

### 4.5. Contribution Measurement Algorithm Based on the Shapley Value

The core idea of our algorithm is that, in each epoch, the server collects local models from different participants, uses them to calculate the relevant gradient information, and uses the gradient information to approximately reconstruct the model on different dataset combinations, avoiding additional training. Next, we use Formula (3) to evaluate the performance of these reconstructed models and further evaluate the contribution level $\phi_{i}$ of each participant. Ultimately, we obtain the final result by taking a weighted average of $\phi_{i}$ values from different training epochs. See the pseudo-code description for details. Algorithm 1 is divided into two parts. Lines 1–22 depict the server-side execution. More specifically, lines 1–13 illustrate the calculation process for the global model: The server first initializes a global model and sends it to each participant. Participants use their local data to perform local training on the initial model sent by the server and upload the trained local model and local class distribution differences to the server. Upon receiving this information, the server calculates each participant’s gradient information $\Delta {\left(t+1\right)}_{i}$ and stores this gradient information along with the cycle information for subsequent global model updates. Then, using $F_{k}$, it calculates the aggregation weights with distribution differences and updates the global model. In lines 14–19, leveraging Formula (3) and the reconstructed model from each epochs, we estimate $\phi_{i}^{\left(t+1\right)}$, representing the contribution index of participant i in epoch t + 1. In line 21, we use the attenuation factor ω∈ (0,1) to regulate the calculation of the final SV. The purpose is that, when calculating the global SV, it is gradually affected by the common influence of all datasets due to increasing iteration epochs. Therefore, earlier epochs are given a higher weight. Lines 23–32, the second segment, is executed by the client: In the first epoch of model training, participants first calculate the local class distribution information $F_{i}$. They can then calculate the local class distribution difference $F_{k}$ (only in the first epoch of computation). Subsequently, the client receives the global model sent from the server and trains the local model using the gradient descent algorithm with local data. The trained local model and the local class distribution difference $F_{k}$ are then uploaded to the trusted server.
**Algorithm 1** FL participant contribution evaluation.**Input:** B is the local minibatch size, E is the number of local epochs, and η is the learning rate; $F_{G}$ is the global distribution we set.**Server executes:**    1:*N*⟵{1, 2, ··· , n};  2:Initialize $M^{\left(0\right)}$, $\left\{M_{s}^{\left(0\right)}|S\subseteq N\right\}$;  3:$F_{i}$⟵ CalculateLocal$\left(i,D_{i}\right)$ for client *i*∈*N*;  4:**for** each epoch t ⟵ 0, 1, 2⋯, *T*−1 **do**  5: /* Calculate the Global Model */  6: $M_{i}^{\left(t+1\right)}$⟵ LocalUpdate$\left(i,M^{\left(t+1\right)}\right)$ for client *i*∈*N*;  7: $\Delta_{i}^{\left(t+1\right)}$⟵$M_{i}^{\left(t+1\right)}-M^{\left(t\right)}$ for client *i*∈*N*;  8: /* Calculate aggregation weight*/  9: $D_{k}=N_{k}-a*F_{k}+b$ Where $N_{k}=\frac{|D_{i}|}{|D_{N}|}$;10: $M^{\left(t+1\right)}$⟵$M^{\left(t\right)}+\sum_{i\in N}D_{\left(k=i\right)}\Delta_{i}^{\left(t+1\right)}$;11: **for** each subset *S*∈*N* **do**12:  $\Delta_{S}^{\left(t+1\right)}$⟵$\sum_{i\in S}D_{k}\Delta_{i}^{\left(t+1\right)}$;13:  $M_{S}^{\left(t+1\right)}$⟵$M^{\left(t\right)}+\Delta_{S}^{\left(t+1\right)}$;14: **end for**15: /* Calculate the epoch-$\phi_{i}$ */16: **for** i⟵ {1, 2, ··· , *n*} **do**17:  $\phi_{i}^{\left(t+1\right)}=C\cdot \sum_{S\subseteq N\backslash i}\frac{U\left(M_{S\cup i}^{\left(t+1\right)}\right)-U\left(M_{S}^{\left(t+1\right)}\right)}{\left(\begin{array}{c} n-1|S| \end{array}\right)}$;18: **end for**19:**end for**20:/* Calculate the final $\phi_{i}$ */21:$\phi_{i}=\sum_{t=1}^{R}\omega^{t}\cdot \frac{\phi_{i}^{\left(t\right)}}{\sum_{i=1}^{n}\phi_{i}^{\left(t\right)}}$ for client *i*∈*N*; **return** $M^{\left(R\right)}$ and $\phi_{1}$, $\phi_{2}$, ..., $\phi_{n}$;**Client executes:**  23:$F_{k}$⟵ CalculateDistributionDifferences $\left(F_{i},F_{G}\right)$ for client (*i* = *k*) ∈*N* (Only counted in the first epoch) ;24:LocalUpdate(*i*, *M*):25:/* Calculate the Local Model */26:*B*⟵(split $D_{i}$ into batches of size B);27:**for** each local epoch e⟵ 1, 2, ..., *E* **do**28: **for** batch b ∈*B* **do**29:  *M*⟵M−η∇L(M;b);30: **end for**31:**end for**32:**return** *M* and $F_{k}$ to server

## 5. Experiment and Results

In this section, we perform experiments on the aforementioned algorithms under various data distributions to assess their performance and compare them against existing mainstream methods.

### 5.1. Dataset

This experiment was conducted on the MNIST [48] and Fashion MNIST [49] datasets. The MNIST dataset consists of a collection of over 60,000 training images and more than 10,000 test images of handwritten digits. We randomly sampled 5421 images for each digit category, totaling 54,210 samples for the experiment. Additionally, we randomly selected 8920 images for each digit as a separate test dataset, resulting in a total of 89,200 samples for testing. The Fashion MNIST dataset is a clothing classification dataset containing 10 categories, with the same amount of training and test data as the MNIST dataset. Compared to MNIST, Fashion MNIST presents higher challenges in terms of image quality and diversity, as it includes more backgrounds and different perspectives. We performed the same data sampling process on the Fashion MNIST dataset to form the training and test sets used in our experiments to meet our requirements.

For the FL experiment, we established five clients; this is a relatively common setup. In fact, our algorithm remains applicable and even has greater advantages when there are more participants involved. In scenarios with a large number of participants, existing methods to evaluate the SV (Shapley value) of the participants often rely on sub-model retraining. If there are many participants, the reconstruction of the model involves extensive training, which requires a lot of participants to retrain a sub-model, consuming significant computational resources and increasing the model’s runtime. Our model reconstructs the model based on gradients, eliminating the need for sub-model retraining. The more participants there are, the greater the advantage for our model, as our runtime will be significantly reduced. For servers with limited computational resources, our method can greatly alleviate the computational burden on the server compared to other methods in scenarios with a large number of participants. To evaluate the performance of the proposed algorithm under different FL settings, we set up five different federated scenarios for testing:

- **Same Distribution and Same Size:** In this setup, we randomly partitioned the dataset into five equally sized subsets, each containing the same number of images and maintaining the same label distribution.
- **Same Distribution and Different Size:** We extracted the data we intended to use from the training set and divided them into 20 portions to create a local dataset for each participant. The proportion for participant 1 is 2/20; for participant 2, it is 3/20; for participant 3, it is 4/20; for participant 4, it is 5/20; and for participant 5, it is 6/20. We ensured that the amount of data varied between participants, and within each participant’s dataset, each numerical category had the same quantity.
- **Different Distributions and Same Size:** In this setup, we divide the dataset into five equal parts, with each part having a distinct distribution of feature data. To be specific, the dataset for participant 1 comprises 80% of the samples labeled as “1” and “2”, while the remaining 20% of the samples are equally distributed among the other numbers. This distribution strategy also applies to participants 2, 3, and 4. The final client exclusively contains handwritten numeric samples labeled as “8” and “9”.
- **Biased and unbiased:** This method builds upon the “Different Distributions and Same Size” method by aiming to enhance the heterogeneity of data distributions among clients. Under the condition that each participant possesses an equal number of samples, the method employs a more heterogeneous setup. It includes four biased clients, each containing two categories of non-overlapping data, and one unbiased client with an equal number of samples from all ten categories.
- **Noisy Labels and Same Size:** The data partitioning in this setting is identical to that in the “Same Distribution and Same Size” method. Subsequently, varying proportions of Gaussian noise are introduced to the input images. The specific configuration is as follows: participant 1 has 0% Gaussian noise; participant 2 has 5%; participant 3 has 10%; participant 4 has 15%; and participant 5 has 20%.

### 5.2. Baseline Algorithm

Although there are many federated learning contribution measurement schemes based on the Shapley value, they often involve additional model evaluation and affect model convergence. Therefore, we selected the widely used and classic contribution measurement scheme as the baseline algorithm. These schemes employ measures to reduce the need for additional model evaluation, and our work is more appropriate in comparison with these schemes. Here are the algorithms we used to make the comparison:

- **Exact Shapley:** This is the exact computation method proposed in the literature [32]. This method calculates the original Shapley values according to Formula (3), which involves a large amount of sub-model reconstruction. It evaluates all possible combinations of participants, and each sub-model is trained using their respective datasets.
- **TMC Shapley:** The method mentioned in the literature [32] is the truncated Monte Carlo Shapley algorithm, which uses local datasets and the initial FL model to train models for a subset of FL participants. To reduce unnecessary computational resources, the Monte Carlo Shapley value estimation is achieved by randomly sampling permutations and truncating unnecessary sub-model training and evaluation. Specifically, during model training, in each iteration, the algorithm generates a random sequence of training data points. The performance of the model trained with the first j datasets of the current random permutation is compared to the performance of the model obtained using all training datasets. If the difference is less than a predefined performance tolerance, it indicates that the addition of subsequent datasets will not produce new marginal contributions, and further model training is not required. Otherwise, the model needs to be retrained with the first j datasets to obtain new model performance.
- **K-subset Shapley:** This method [34] randomly takes every possible size subset of a participant, strictly records the occurrence time of size K, and reconstructs the participant’s Shapley value to his expected contribution to a K-size subset with a random base. The way retains the hierarchical structure of the Shapley value, and it has high approximation precision.
- **SOR Shapley:** Similar to the OR method in the literature [7], this method uses gradients to reconstruct sub-models, thereby avoiding the need for local users to retrain and thus saving computational resources. The Shapley value for each participant is calculated at each training epoch, and the results are recorded. These contribution results are then aggregated to reflect the overall performance of each client in federated learning.
- **TMR Shapley:** This method is the truncated multi-round (TMR) construction introduced in [35], which is an improvement of the MR algorithm. It uses a decay factor λ and the accuracy of each round to control the weights of the round-level $\phi^{\left(t\right)}$ in the final result. Once the round-level $\phi^{\left(t\right)}$ become negligible for the final result, the model is no longer constructed or evaluated. Specifically, during the iterative process of federated learning, when calculating each participant’s round-level $\phi^{\left(t\right)}$, we check whether $\lambda^{t}$ is less than the threshold we set (at this point, round-level $\phi^{\left(t\right)}$ can be considered negligible for the final result). If it is, the contribution assessment is truncated, and its result is not included in the final calculation of the participant’s contribution. This approach saves computation time and improves efficiency.

At the same time, we analyzed the baseline algorithm used in the experiment and the algorithm proposed by us, including the technology adopted by the algorithm, time complexity, advantages and disadvantages, etc. The specific details can be seen in Table 1.

### 5.3. Performance Evaluation Metrics

The performance of the comparison approaches is evaluated with the following metrics:

- **Time:** We compared the training time of the model with the time required to calculate the contribution index.
- **SV:** We compare the Shapley value of the participants obtained using different algorithms in various scenarios.
- **Accuracy:** We evaluated the model accuracy using different algorithms in various scenarios.

### 5.4. Hyper-Parameters Setting

Through experiments, we studied the effect of different values of a and b on the performance of the model to determine the ideal weights for our experiment. We studied the performance of the model with *a* and *b* on different datasets in the same federated scenario. As shown in Table 2 and Table 3, when a=0.6 and b=0.1, our model achieves the best gain, which means the optimal aggregation weight for the participants can be obtained.

### 5.5. Experimental Result

In this section, we conduct experiments on several federated scenarios with different datasets and analyze the experimental results.

#### 5.5.1. Experimental Result on MNIST

**Figure 4:**
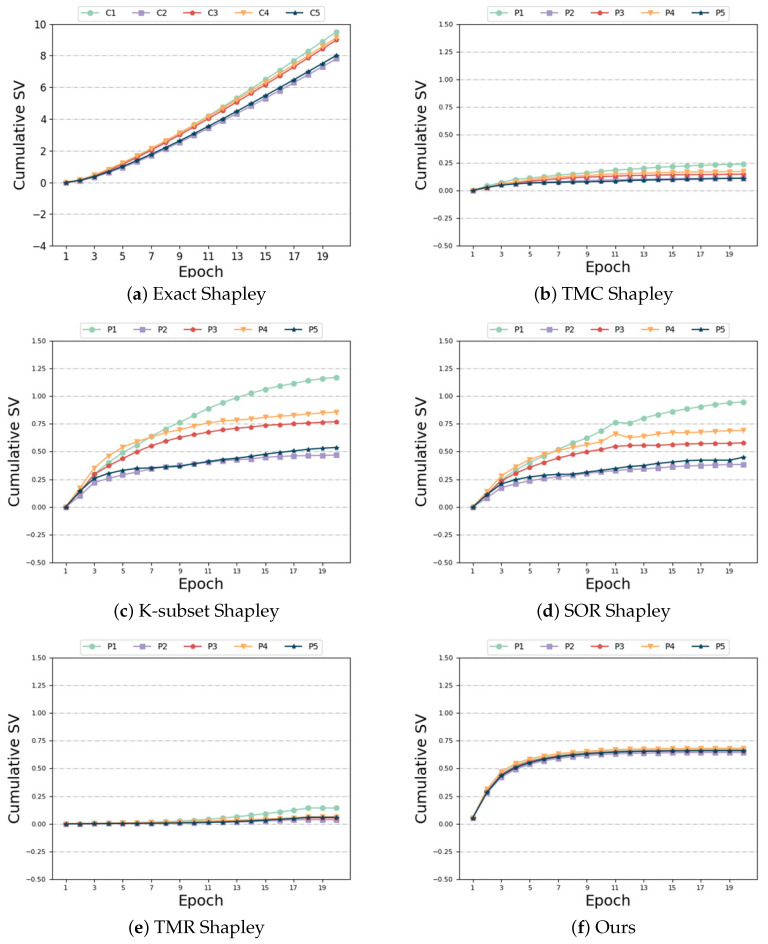
Same distribution and same size.

Same distribution and same size: In this case, each participant is assigned to the same data category and quantity. Thus, the expected outcome is for each participant to have the same SV. Figure 4 illustrates the variations of different algorithms with respect to the number of training epochs in this scenario. As observed in Figure 4a,c,d, the performance of “Exact Shapley”, “K-subset Shapley”, and “SOR Shapley” methods is not optimal, resulting in significant variation in SV among the five participants. Figure 4b,e,f demonstrate that, although the “TMC Shapley” and “TMR Shapley” algorithms achieved the desired outcome with relatively close SV among multiple participants, their performance is still inferior to our algorithm. The results obtained from our algorithm indicate that the SV of the five participants are very close to one another. Regarding time and accuracy, Table 4 reveals that the “Exact Shapley” method exhibits low efficiency, requires substantial time, and yields subpar model accuracy. The “TMR Shapley” algorithm takes the least time, but its model accuracy is lower than ours. Our method attains favorable outcomes while maintaining comparable runtime to other baseline algorithms.

**Figure 5:**
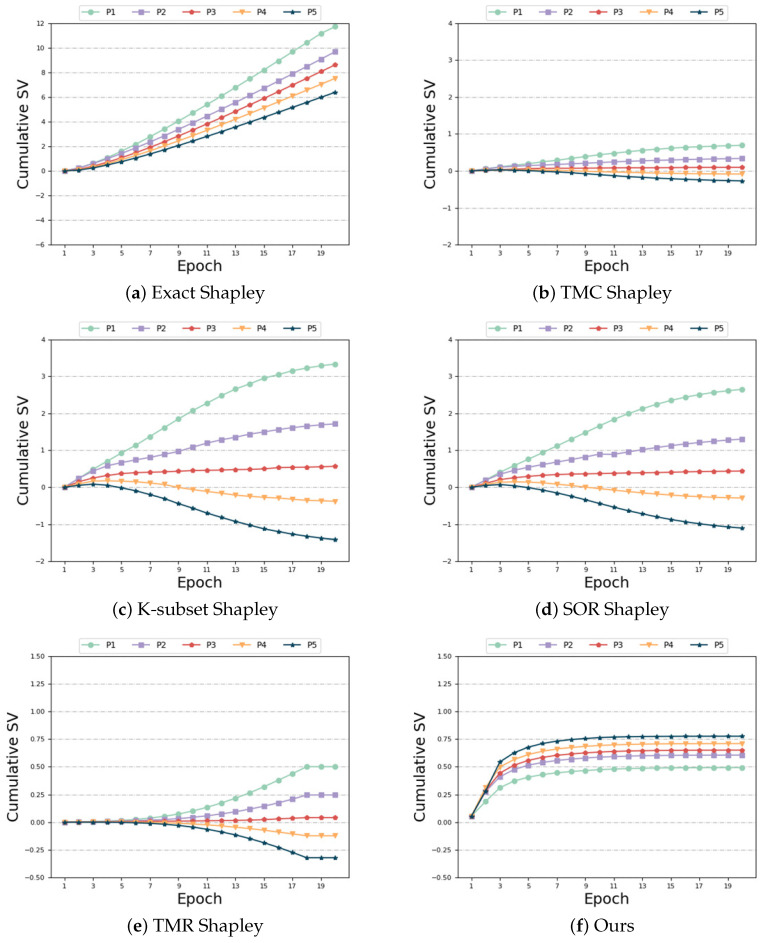
Same distribution and different size.

Same distribution and different size: In this setting, we make the feature distribution of the participants consistent and change the number of their features. From Formula (2), we can see that the more characteristic data the player has, the greater the weight, and theoretically the greater SV. However, as can be seen from Figure 5a–e, the baseline algorithms are ill-suited for this non-IID scenario, resulting in unsatisfactory experimental outcomes. When the last participant possesses the most feature data, their contribution should far exceed that of other participants. Furthermore, the SV of the first four participants exhibit a decreasing trend, contrary to the actual results. Figure 5f demonstrates that our algorithm effectively captures this characteristic, yielding precise measurement results. Our algorithm accurately determines that the participant (Participant 5) with the highest number of feature data has a positive and maximum SV. In addition, it correctly captures the increasing trend of the SV among the first four participants, unlike the unreasonable measurement results of the baseline algorithm. In terms of time and accuracy, we can see from Table 5 that, although our time cost is not optimal, it is still within the acceptable range, but compared with other baseline algorithms, we have higher accuracy. Compared with the “Exact Shapley” algorithm, the algorithm time is greatly reduced.

**Figure 6:**
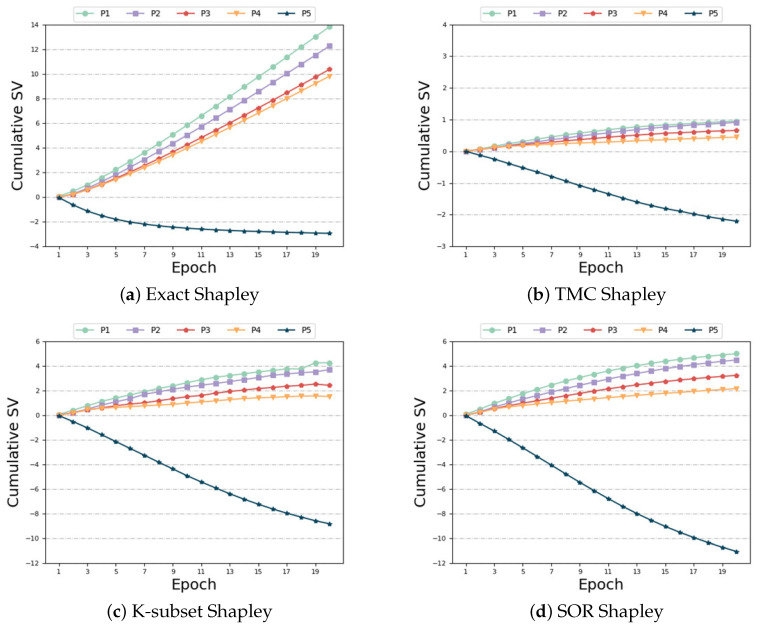

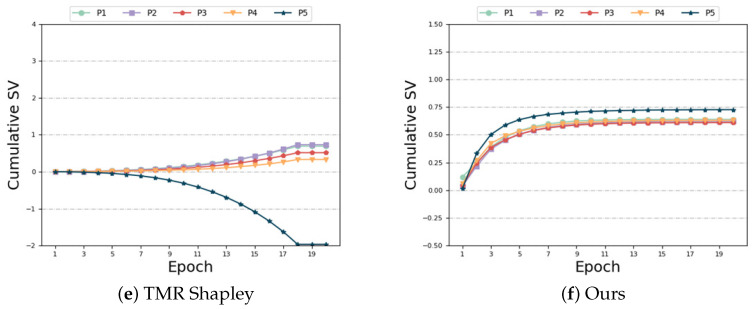
Different distributions and same size.

Different distributions and same size: In this setting, each participant is allocated the same data quantity, but their feature distributions are heterogeneous. First, let us look at the SV measurement. As depicted in Figure 6a–e, the following observations can be made: Among the compared baseline algorithms, none of them exhibit robustness against the heterogeneity of the data distribution. Specifically, when participant 5’s data exhibits high heterogeneity, the baseline method yields a negative SV for that participant. However, this result contradicts the definition of SV. Participants who contribute high-quality data through realistic training should be assigned larger SV, whereas those providing random data should receive smaller SV [50]. The presence of heterogeneous data adversely affects the accuracy of SV measurements. Our algorithm alleviates the impact of heterogeneous data and accurately assigns a positive SV to participant 5, as evident from Figure 6f. In contrast to the baseline algorithm, our algorithm achieves accurate measurement of the SV for each participant. As evident from Table 6, the “Exact Shapley” method remains computationally expensive. Although the time of “TMR Shapley” algorithm is shorter, the model accuracy is not as good as ours. Our algorithm achieves better results when the time overhead is similar to other baseline algorithms.

**Figure 7:**
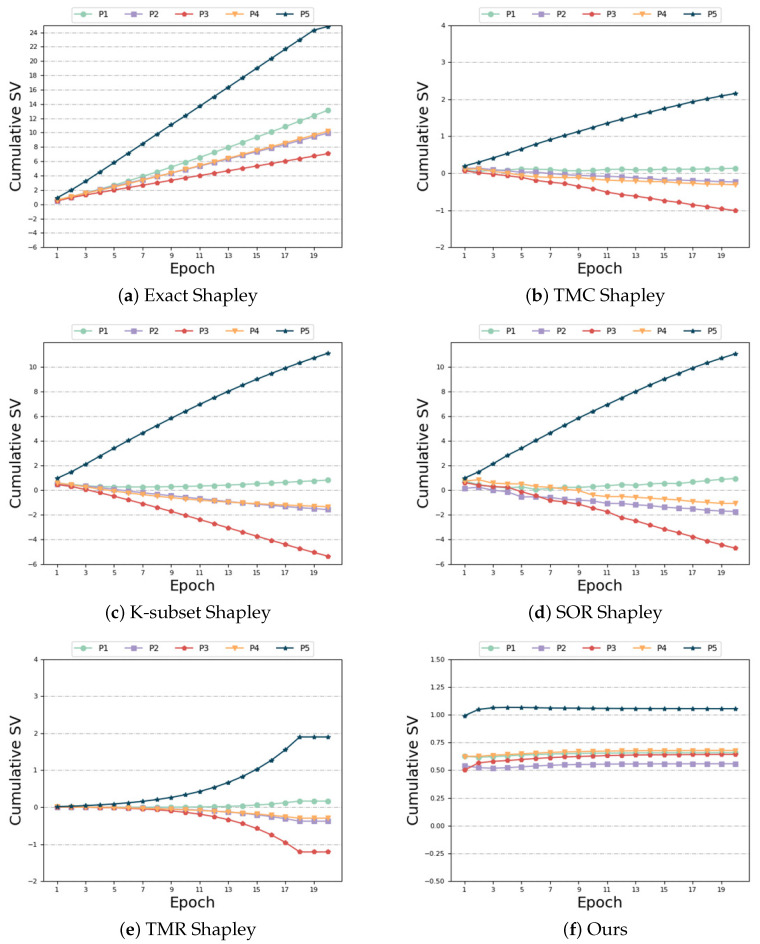
Biased and unbiased.

Biased and unbiased: This setting is similar to the “Different Distributions and Same Size” scenario, but it exhibits a greater degree of heterogeneity in feature distribution. Figure 7 provides additional evidence of our algorithm’s robustness in handling data distribution heterogeneity. Although all the algorithms measured a higher contribution from the last unbiased participant, the outcomes for the remaining four biased participants were unsatisfactory. Figure 7b–e demonstrate that the SV of the four biased participants, as measured by the “TMC Shapley”, “K-subset Shapley”, “SOR Shapley”, and “TMR Shapley” methods, are negative. Figure 7a,f illustrate that the “Exact Shapley” method assigns a positive SV to participant 5, but it exhibits significant discrepancies in SV measurements for the four biased participants. Our algorithm accurately captures the larger SV of the unbiased participant while assigning the smaller positive SV to the remaining four biased participants who have no harmful data and achieves subtle numerical differences between the biased participants. This is consistent with expectations and improves accuracy compared to the baseline algorithm. Table 7 highlights that the “Exact Shapley” method incurs the highest time overhead, and the “TMR Shapley” algorithm still has the best time overhead. Our algorithm exhibits comparable time requirements to other baseline algorithms, while achieving superior accuracy in this scenario.

**Figure 8:**
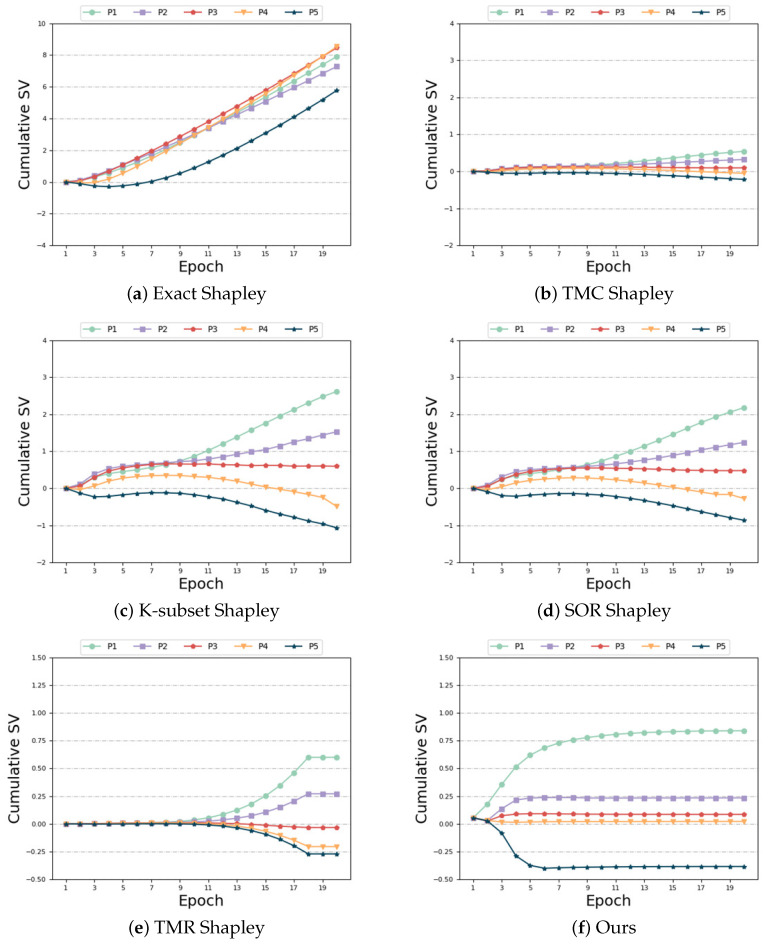
Noise labels and same size.

Noise labels and same size: Next, we investigate the impact of noise labels, as illustrated in Figure 8. Figure 8b–f demonstrate the robustness of our algorithm and the baseline algorithms (“TMC Shapley”, “K-subset Shapley”, “SOR Shapley”, and “TMR Shapley”) to noisy data. Specifically, the SV of the last four participants with noisy data exhibits the expected decreasing trend, distinctly differing from the SV of the first participants with noiseless data. The SV of the last four noisy players is much smaller than the SV of the first noiseless player. However, in the case of the “Exact Shapley” algorithm, Figure 8a reveals that the SV of the last four participants with noisy data do not show the expected decreasing trend, and the SV of Participant 1 without noise is smaller than that of participants with noisy data. Consequently, the results are evidently inaccurate. Regarding time and accuracy, Table 8 indicates that the “Exact Shapley” algorithm incurs significant time overhead without achieving high model accuracy. Our algorithm achieves model accuracy comparable to other baselines, and, although the time overhead is not optimal, it is still within the acceptable range.

#### 5.5.2. Experimental Result on Fashion-MNIST

We selected the three most common scenarios from the previously set federated scenarios to conduct experiments, in order to validate the performance of our algorithm on the Fashion-MNIST dataset and analyze the experimental results.

**Figure 9:**
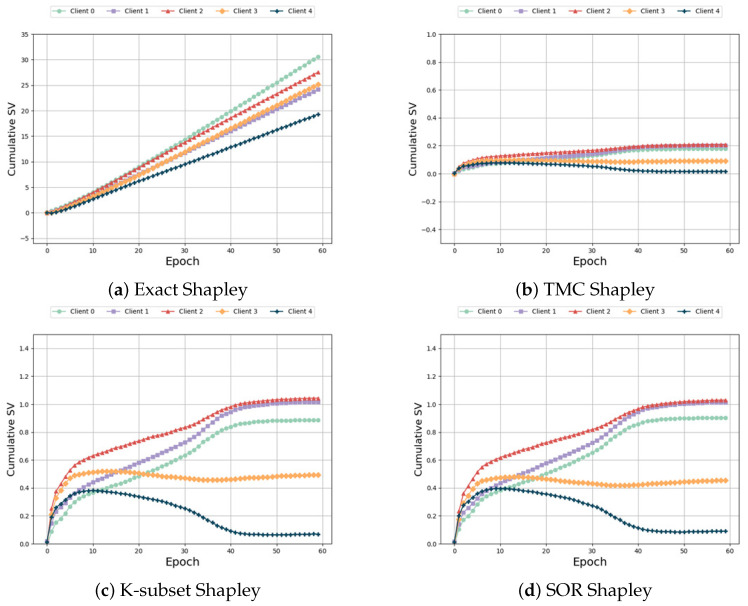

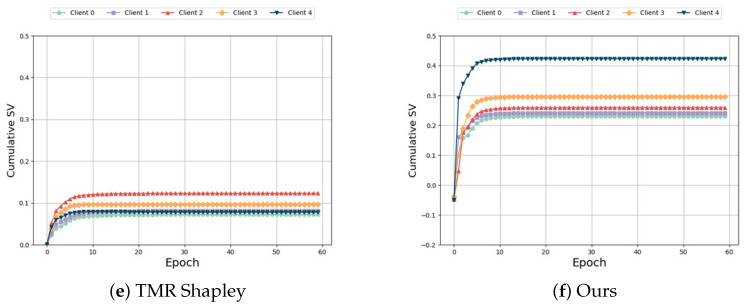
Same distribution and different size.

Same distribution and different size: From Figure 9, it can be seen that in this federated scenario, due to the proportionality of the data among the five participants, according to the definition in Formula (2), participants with larger amounts of data are allocated greater weights and theoretically have higher SV. However, due to the issue of data heterogeneity, the experimental results did not meet expectations. From Figure 9a–e we can observe that the participant with the most feature data, Participant 5, received the lowest SV in the baseline algorithm. As shown in Figure 9f, our algorithm achieves better results by adjusting the aggregate weights of the participants: the SV of the last participant is larger than the SV of the first four participants. As shown in Table 9, although our algorithm does not have the optimal runtime, it achieves higher accuracy compared to the baseline algorithm.

**Figure 10:**
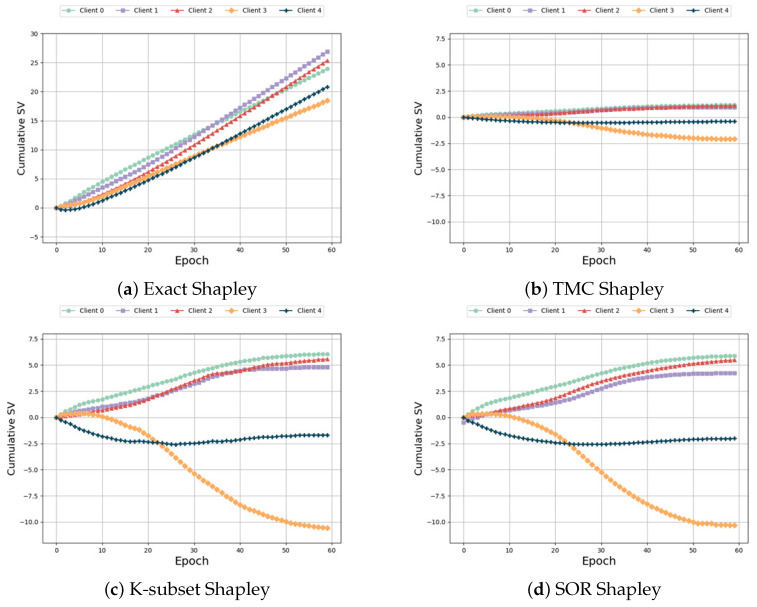

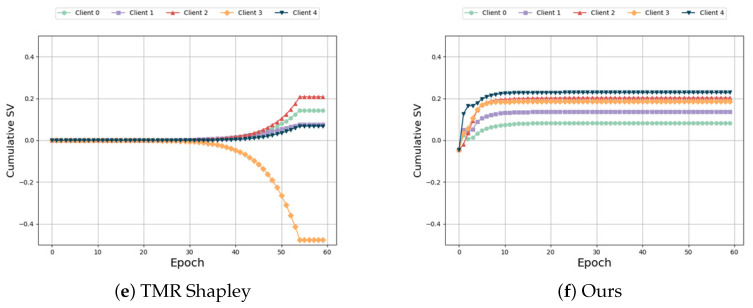
Different distributions and same size.

Different distributions and same size: From Figure 10a–e, it can be seen that in this federated scenario, when the last participant has highly heterogeneous data, the gradient information it uploads affects the performance of the global model, causing participant 5 to show poor performance on the baseline algorithm or even have a negative impact (negative SV). From Figure 10f, it can be seen that the performance of our algorithm remains consistent with its performance on MNIST. Our algorithm adjusts its aggregation weight to mitigate the impact of the heterogeneous data, thereby measuring its SV more accurately. As shown in Table 10, our algorithm achieves higher accuracy compared to the baseline algorithm, and the runtime of the algorithm is also reasonable.

**Figure 11:**
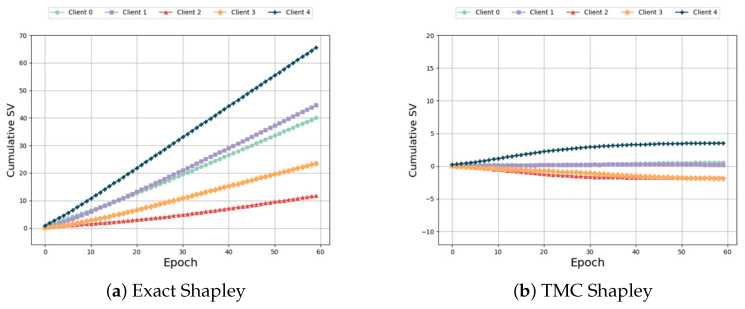

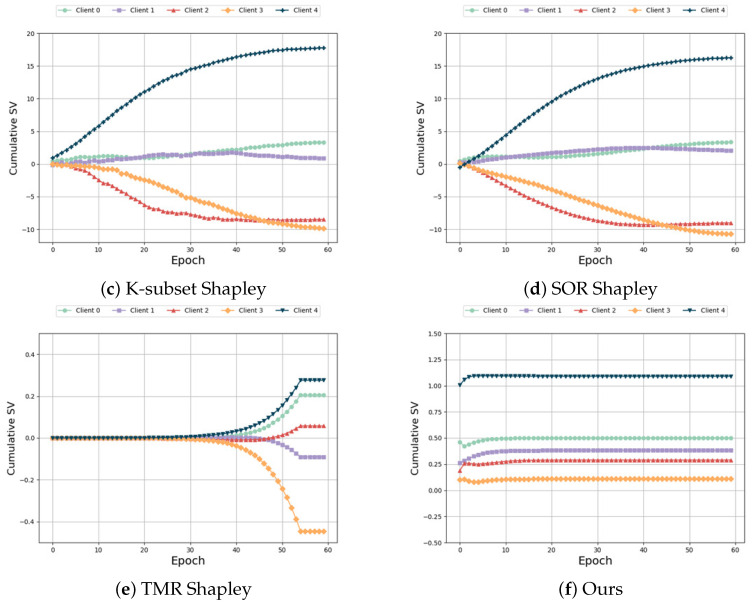
Biased and unbiased.

Biased and unbiased: From Figure 11, it can be seen that in this federated scenario, both our algorithm and the baseline algorithm demonstrate a certain level of robustness: the first four participants have high data heterogeneity and thus have lower SV, whereas the last participant has uniformly distributed data and, consequently, a much higher SV. However, since there is no significant difference in the degree of heterogeneity among the first four participants, their SV should be approximately similar. However, in the baseline algorithm, the SV of the first four participants vary greatly, and the results are not as accurate as those measured by our algorithm in Figure 11f. As shown in Table 11, the Exact-Shapley algorithm takes the most time and is less efficient. Our algorithm, with a reasonable runtime, achieves the highest accuracy compared to all baseline algorithms.

## 6. Conclusions

This paper proposes a fair contribution evaluation method based on the Shapley value, addressing the limitations of existing federated learning (FL) contribution evaluation methods. The proposed method assesses participants’ contributions to the FL model performance based on the Shapley value, without requiring additional model training or exposing sensitive local data. Its key idea involves reconstructing the model using gradients. Additionally, we utilize a novel aggregation function to address the issue of data distribution heterogeneity, thereby mitigating its impact on the measurement of contributions. Extensive experiments were conducted on the MNIST dataset, demonstrating that our method accurately measures participant contributions and exhibits robustness for non-IID data. Our method achieves comparable speed and higher accuracy compared to other baseline approaches.

However, the algorithm’s performance is suboptimal when dealing with noisy label data. In future work, attention mechanisms could be incorporated to enable the model to selectively attend to the noiseless components, thereby mitigating the impact of noisy labels. We can build a deep learning model with an attention mechanism on each client, such as a convolutional neural network (CNN) or a recurrent neural network (RNN), and add an attention layer. We can integrate the attention mechanism in the model to allow the model to automatically learn and focus on important features, thereby reducing the impact of noisy labels. Each client uses its own data (containing noisy labels) to train the model and reduces the noise impact through the attention mechanism to generate local model updates. Furthermore, this method holds potential for extension to the domain of federated medical image segmentation. In the medical field, due to concerns about privacy issues, all parties are reluctant to participate in federated learning. Although federated learning can solve privacy problems, in the field of federated medical image segmentation, it also faces the non-IID problem (the same disease is caused by different factors, but the manifestations of the lesion will be different, showing obvious regional characteristics), resulting in unsatisfactory segmentation results. Expanding our method in the field of image segmentation can effectively reduce the impact of the non-IID problem and improve the accuracy of model segmentation.

## Figures and Tables

**Table 1:** Data evaluation approaches based on Shapley value.

Name	Time Complexity	Approach	Characteristics
Exact Shapley [32]	$O(N^{2}\log N)$	Submodel reconstruction	Low computation complexity, time-consuming
TMC Shapley [32]	$O(N^{2}\log N)$	Truncation, model approximation	Reduced computation, high error risk
K-sub Shapley [34]	$O(N^{2}\log N)$	Stratified sampling	Reduced computation, Loss of some precision
SOR Shapley [7]	$O(N^{2}\log N)$	Model approximation	Reduced computation, unnecessary estimation
TMR Shapley [35]	$O(N^{2}\log N)$	Truncation, model approximation	Truncation reduces computation, high error risk
Ours	$O(N^{2}\log N)$	Reconstructed model based on gradient	Reduced computation, mitigate the effects of Non-IID, lack of noise data sensitivity

**Table 2:** MNIST, different distributions and same size, exact Shapley (FedAVG). The optimal results have been shown in bold in the table.

a\b	0.05	0.1	0.2	0.3	0.4
0.2	0.56	0.03	0.20	0.32	0.63
0.3	0.43	0.74	0.39	0.31	0.12
0.4	0.21	1.09	0.14	0.22	0.16
0.5	0.72	1.29	0.57	0.20	0.89
0.6	0.17	**2.54**	0.77	0.66	1.39
0.7	0.61	0.76	0.71	1.19	0.06

**Table 3:** Fashion-MNIST, different distributions and same size, exact Shapley (FedAVG). The optimal results have been shown in bold in the table.

a\b	0.05	0.1	0.2	0.3	0.4
0.2	0.74	0.27	0.06	0.79	0.19
0.3	0.39	0.89	0.24	0.89	0.27
0.4	0.53	0.49	0.52	0.35	0.59
0.5	0.49	0.19	0.69	0.45	0.69
0.6	0.77	**1.97**	0.71	0.58	0.56
0.7	0.89	0.54	0.83	0.83	0.19

**Table 4:** Time and accuracy.

Name	Time	Accuracy
Exact Shapley [32]	10,840.24 s	84.93%
TMC Shapley [32]	720.56 s	86.95%
K-subset Shapley [34]	601.76 s	86.87%
SOR Shapley [7]	636.94 s	86.70%
TMR Shapley [35]	574.05 s	86.69%
Ours	646.35 s	90.64%

**Table 5:** Time and accuracy.

Name	Time	Accuracy
Exact Shapley [32]	11,570.86 s	85.43%
TMC Shapley [32]	737.58 s	86.71%
K-subset Shapley [34]	630.42 s	86.32%
SOR Shapley [7]	600.94 s	86.83%
TMR Shapley [35]	587.43 s	86.72%
Ours	729.98s	88.38%

**Table 6:** Time and accuracy.

Name	Time	Accuracy
Exact Shapley [32]	10,865.56 s	84.42%
TMC Shapley [32]	659.58 s	85.53%
K-subset Shapley [34]	679.24 s	85.72%
SOR Shapley [7]	695.14 s	85.83%
TMR Shapley [35]	564.35 s	85.68%
Ours	655.78 s	86.96%

**Table 7:** Time and accuracy.

Name	Time	Accuracy
Exact Shapley [32]	10,961.56 s	83.25%
TMC Shapley [32]	650.53 s	82.98%
K-subset Shapley [34]	662.89 s	83.32%
SOR Shapley [7]	637.49 s	82.83%
TMR Shapley [35]	587.93 s	82.51%
Ours	655.78 s	84.39%

**Table 8:** Time and accuracy.

Name	Time	Accuracy
Exact Shapley [32]	143,196.81 s	78.85%
TMC Shapley [32]	784.17 s	79.12%
K-subset Shapley [34]	630.42 s	79.26%
SOR Shapley [7]	625.95 s	78.90%
TMR Shapley [35]	565.53 s	78.94%
Ours	716.84 s	80.86%

**Table 9:** Time and accuracy.

Name	Time	Accuracy
Exact Shapley [32]	32,517.0 s	78.29%
TMC Shapley [32]	1546.66 s	78.69%
K-subset Shapley [34]	1286.26 s	79.37%
SOR Shapley [7]	1185.95 s	79.51%
TMR Shapley [35]	1424.55 s	79.78%
Ours	2118.53 s	81.45%

**Table 10:** Time and accuracy.

Name	Time	Accuracy
Exact Shapley [32]	22,376.16 s	77.18%
TMC Shapley [32]	1267.93 s	78.23%
K-subset Shapley [34]	1228.16 s	77.95%
SOR Shapley [7]	1465.27 s	78.11%
TMR Shapley [35]	1535.23 s	78.18%
Ours	1701.67 s	79.15%

**Table 11:** Time and accuracy.

Name	Time	Accuracy
Exact Shapley [32]	19,426.56 s	78.55%
TMC Shapley [32]	984.67 s	79.72%
K-subset Shapley [34]	971.39 s	79.64%
SOR Shapley [7]	938.56 s	79.67%
TMR Shapley [35]	1058.56 s	79.28%
Ours	2058.26 s	80.82%

## Data Availability

Data are contained within the article. Code is available at https://github.com/Guopenghaha/Contribution-Measurement.git.
